# Supplementary material for: Engineering a single-chain immunoglobulin scaffold loaded with a latent-releasable cytotoxic pore-forming peptide
Source: Commun Biol. 2025 Nov 25;8:1665. doi: 10.1038/s42003-025-09066-9 (PMC12647727; doi:10.1038/s42003-025-09066-9)
Supplement: Supplementary file 1 — Supplementary information [file 42003_2025_9066_MOESM1_ESM.pdf]

Supplementary Information

Engineering a single-chain immunoglobulin scaffold loaded with a latent-releasable cytotoxic pore-forming peptide

Izaskun Morillo[a], Joao Zulaica[a,b], Asier R. Caballero[a], Jaione Auzmendi-Iriarte[c], Eneko Largo[d], Beatriz Apellaniz[e,f], Arkaitz Carracedo[b,c,g,h,i], Marco Piva[c,g,h], José L. Nieva[a,b,*], and Edurne Rujas[a,f,h,j,*]

[a]Instituto Biofisika (UPV/EHU, CSIC), University of the Basque Country, Leioa 48940, Spain.

[b]Department of Biochemistry and Molecular Biology, University of the Basque Country (UPV/EHU), Bilbao 48080, Spain.

0

[c]CICbioGUNE, 48160 Derio, Spain.

[d]Department of Immunology, Microbiology and Parasitology, Faculty of Medicine and Nursing, University of the Basque Country (UPV/EHU), 48940 Leioa, Spain.

[e]Department of Physiology, Faculty of Pharmacy, University of the Basque Country (UPV/EHU), 01006 Vitoria-Gasteiz, Spain.

[f]Bioaraba Health Research Institute, Microbiology, Infectious Disease, Antimicrobial Agents, and Gene Therapy, 01006 Vitoria-Gasteiz, Spain.

[g]Traslational prostate cancer Research lab, CIC bioGUNE-Basurto, Biocruces Bizkaia Health Research Institute, 48160 Derio, Spain.

[h]Ikerbasque, Basque Foundation for Science, 48013 Bilbao, Spain.

[i]CIBERONC, Madrid, Spain.

[j]Department of Pharmacy and Food Sciences, Faculty of Pharmacy, University of the Basque Country (UPV/EHU), 01006 Vitoria-Gasteiz, Spain.

*Contact authors:

E-mail: edurne.rujas@ehu.eus; joseluis.nieva@ehu.eus

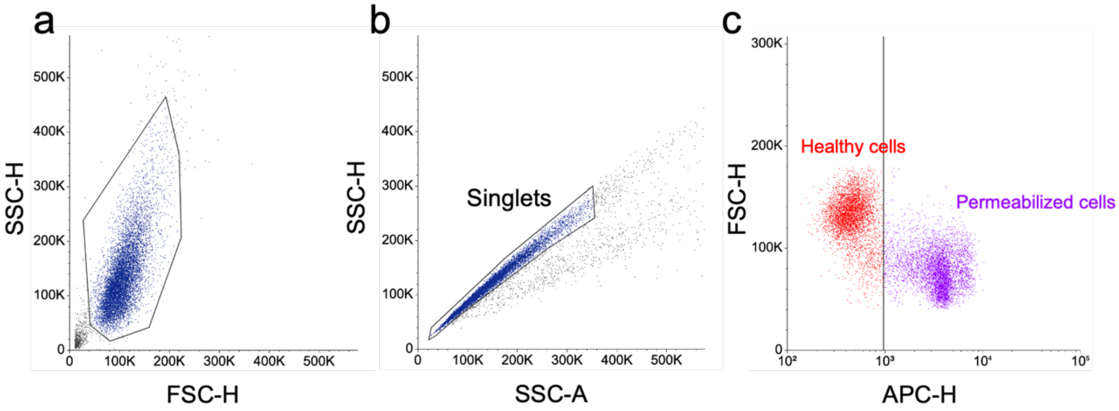

Supplementary Figure 1: Gating strategy in flow cytometry experiments. a) Cells are gated based on FSC vs SSC to select populations with specific size and granularity and **b)** doublets are excluded using SSC-A vs SSC-H gating. **c)** Permeabilized cells stain positive with the 7-AAD probe (detected by APC laser on the cytometer) and exhibit a shift in APC intensity compared to non-permeabilized (healthy) cells. Percentage of permeabilized cells is subtracted from this plot for each condition.

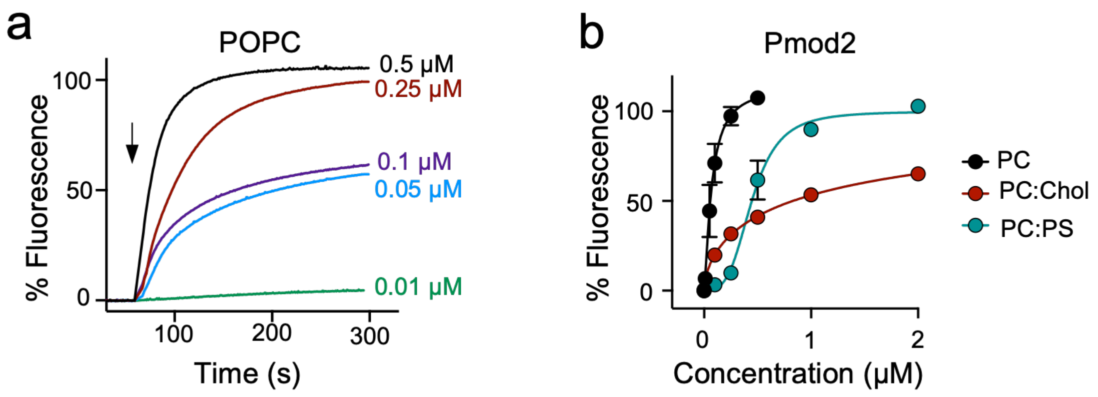

Supplementary Figure 2: Dose-dependent content leakage induced by Pmod2. **a)** ANTS release kinetics following peptide addition (arrow) at increasing concentrations to 100 µM POPC LUVs. **b)** ANTS leakage from POPC (black), POPC:Chol (2:1, red), and POPC:POPS (2:1, cyan) vesicles measured five minutes after peptide addition, plotted as a function of peptide concentration. Mean values ± SD of three independent replicates is shown.

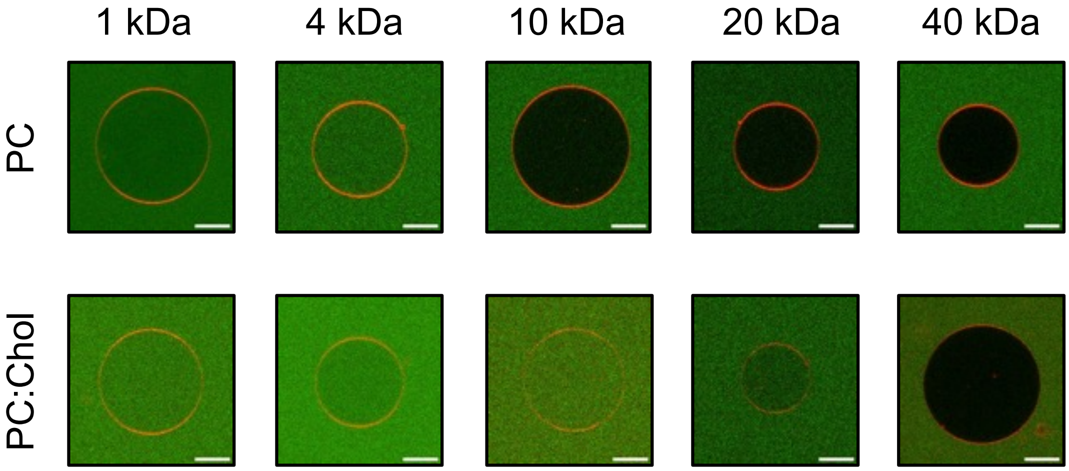

Supplementary Figure 3: Representative confocal micrographs from the single-vesicle permeabilization assay. Peptide-induced permeabilization of POPC (top) and POPC:Chol (bottom) vesicles (shown in orange due to the presence of Rho-PE lipid) is indicated by green fluorescence within the vesicles, resulting from the introduction of AlexaFluor 488 (1 kDa) or FITC conjugated to dextrans of various sizes (4-40 kDa). Scale bar 10 μm.

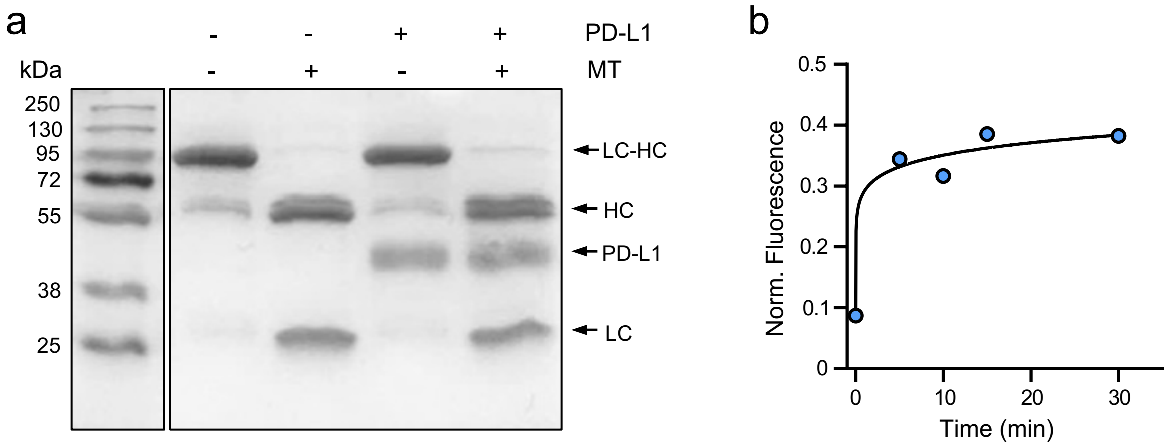

Supplementary Figure 4: MT-induced proteolysis of the scAtezo-Pmod2-2 hybrid. a) SDS-PAGE analysis of the scIgG-Pmod2-2 hybrid incubated for 1 hour with or without MT, in the presence or absence of recombinant PD-L1 ligand. The positions of the LC, HC digested, and linked scIgG (LC-HC) fragments, as well as PD-L1, are indicated by arrows. **b)** ANTS release from POPC LUV upon incubation of the scIgG-Pmod2-2 hybrid with the MT for different incubation times.

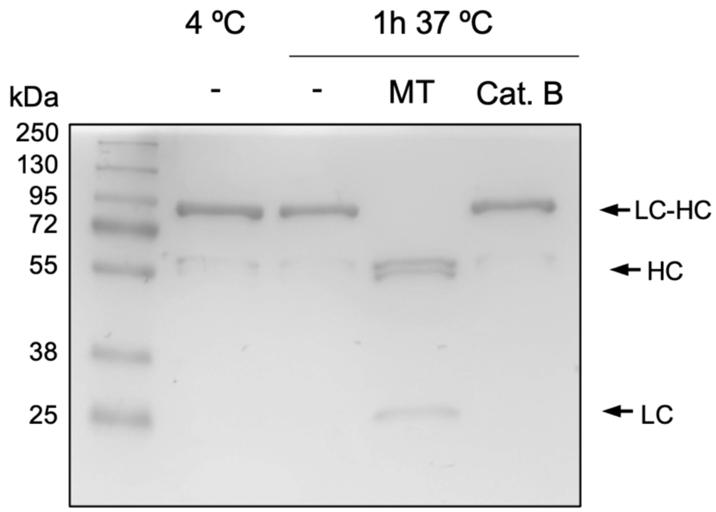

Supplementary Figure 5: Specific cleavage of scAtezo-Pmod2-2. SDS–PAGE analysis of the scIgG-Pmod2-2 hybrid following 1 h incubation at 37 °C with MT, Cat. B, or without MT (control). Arrows indicate the positions of the digested light chain (LC), heavy chain (HC), and the covalently linked scIgG fragment (LC–HC).

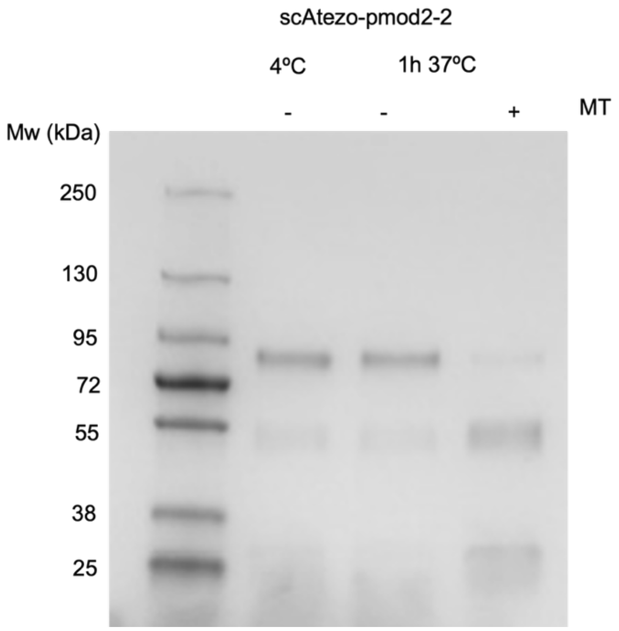

Supplementary Figure 6: Cleavage of scAtezo-Pmod2-2. Uncropped SDS-PAGE gel from figure 6a. SDS–PAGE analysis of the scIgG-Pmod2-2 hybrid after storage at 4 °C and following 1 h incubation at 37 °C with MT or without MT (control).

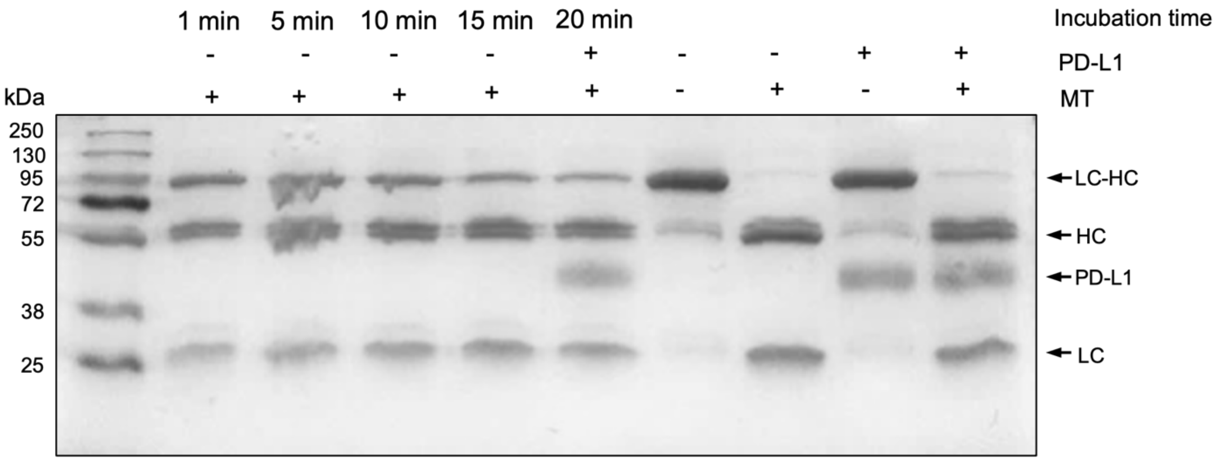

Supplementary Figure 7: Uncropped gel from Supp. Fig. 4a. SDS-PAGE analysis of the scIgG-Pmod2-2 hybrid incubated for different times with or without MT, in the presence or absence of recombinant PD-L1 ligand.
