# Supplementary material for: Engineering a single-chain immunoglobulin scaffold loaded with a latent-releasable cytotoxic pore-forming peptide
Source: Commun Biol. 2025 Nov 25;8:1665. doi: 10.1038/s42003-025-09066-9 (PMC12647727; doi:10.1038/s42003-025-09066-9)
Supplement: Supplementary file 2 — Description of Additional Supplementary Files [file 42003_2025_9066_MOESM2_ESM.pdf]

Description of Additional Supplementary Files 1

File name: Supplementary Data 1

Description: The supplementary data file contains the numerical values and underlying data used to generate all figures presented in the main manuscript. Each dataset is organized according to the corresponding figure and panel, with clear labels indicating experimental conditions, replicates, and measurement units where applicable. This file provides transparency and allows readers to reproduce the analyses and visualizations shown in the study.
