# Supplementary material for: Engineering a single-chain immunoglobulin scaffold loaded with a latent-releasable cytotoxic pore-forming peptide
Source: Commun Biol. 2025 Nov 25;8:1665. doi: 10.1038/s42003-025-09066-9 (PMC12647727; doi:10.1038/s42003-025-09066-9)
Supplement: Supplementary file 4 — Reporting summary [file 42003_2025_9066_MOESM4_ESM.pdf]

nature portfolio

Corresponding author(s): Edurne Rujas

Last updated by author(s): Sep 30, 2025

Reporting Summary

Nature Portfolio wishes to improve the reproducibility of the work that we publish. This form provides structure for consistency and transparency in reporting. For further information on Nature Portfolio policies, see our Editorial Policies and the Editorial Policy Checklist.

Statistics

For all statistical analyses, confirm that the following items are present in the figure legend, table legend, main text, or Methods section.

n/a	Confirmed	
☐	☒	The exact sample size (*n*) for each experimental group/condition, given as a discrete number and unit of measurement
☐	☒	A statement on whether measurements were taken from distinct samples or whether the same sample was measured repeatedly
☐	☒	The statistical test(s) used AND whether they are one- or two-sided<br>*Only common tests should be described solely by name; describe more complex techniques in the Methods section.*
☒	☐	A description of all covariates tested
☒	☐	A description of any assumptions or corrections, such as tests of normality and adjustment for multiple comparisons
☐	☒	A full description of the statistical parameters including central tendency (e.g. means) or other basic estimates (e.g. regression coefficient) AND variation (e.g. standard deviation) or associated estimates of uncertainty (e.g. confidence intervals)
☐	☒	For null hypothesis testing, the test statistic (e.g. *F*, *t*, *r*) with confidence intervals, effect sizes, degrees of freedom and *P* value noted<br>*Give P values as exact values whenever suitable.*
☒	☐	For Bayesian analysis, information on the choice of priors and Markov chain Monte Carlo settings
☒	☐	For hierarchical and complex designs, identification of the appropriate level for tests and full reporting of outcomes
☒	☐	Estimates of effect sizes (e.g. Cohen's *d*, Pearson's *r*), indicating how they were calculated

Our web collection on statistics for biologists contains articles on many of the points above.

Software and code

Policy information about availability of computer code

Data collection	Data acquisition by BLI was done using BLI Discovery version 12.2.Ink
Data analysis	BLI data analysis was performed using Octet analysis studio 12.2.Ink software.<br>Prism (version 10.3.1) was used to generate plots, including cytotoxicity curves, EC_{50} comparison plots, BLI sensograms, and leakage experiment data.<br>Helical wheel representations was done with the Heliquest server available online.<br>Flow cytometry analysis was done using the CytExpert Software

For manuscripts utilizing custom algorithms or software that are central to the research but not yet described in published literature, software must be made available to editors and reviewers. We strongly encourage code deposition in a community repository (e.g. GitHub). See the Nature Portfolio guidelines for submitting code & software for further information.

Data

Policy information about availability of data

All manuscripts must include a data availability statement. This statement should provide the following information, where applicable:

- Accession codes, unique identifiers, or web links for publicly available datasets
- A description of any restrictions on data availability
- For clinical datasets or third party data, please ensure that the statement adheres to our policy

Data used to generate this manuscript are described in Supplementary Data 1. Reagents and additional information are available from the corresponding author upon reasonable request.

Research involving human participants, their data, or biological material

Policy information about studies with human participants or human data. See also policy information about sex, gender (identity/presentation), and sexual orientation and race, ethnicity and racism.

Reporting on sex and gender	n/a
Reporting on race, ethnicity, or other socially relevant groupings	n/a
Population characteristics	n/a
Recruitment	n/a
Ethics oversight	n/a

Note that full information on the approval of the study protocol must also be provided in the manuscript.

Field-specific reporting

Please select the one below that is the best fit for your research. If you are not sure, read the appropriate sections before making your selection.

☒ Life sciences ☐ Behavioural & social sciences ☐ Ecological, evolutionary & environmental sciences

For a reference copy of the document with all sections, see nature.com/documents/nr-reporting-summary-flat.pdf

Life sciences study design

All studies must disclose on these points even when the disclosure is negative.

Sample size	For binding kinetics: two replicates were performed. Since both replicate values differ between each other less than 2 fold we were confident with the value and hence we did not increase the number of replicates further. For cell viability experiments at least three individual experiments have been performed.
Data exclusions	No data were excluded from the analyses.
Replication	All attempts at replication were successful and we have performed three biological replicates.
Randomization	No randomization was necessary for our biophysical and functional studies.
Blinding	No blinding was necessary for our biophysical studies and functional studies.

Reporting for specific materials, systems and methods

We require information from authors about some types of materials, experimental systems and methods used in many studies. Here, indicate whether each material, system or method listed is relevant to your study. If you are not sure if a list item applies to your research, read the appropriate section before selecting a response.

Materials & experimental systems

n/a	Involved in the study
☐	☒ Antibodies
☐	☒ Eukaryotic cell lines
☒	☐ Palaeontology and archaeology
☐	☒ Animals and other organisms
☒	☐ Clinical data
☒	☐ Dual use research of concern
☒	☐ Plants

Methods

n/a	Involved in the study
☒	☐ ChIP-seq
☐	☒ Flow cytometry
☒	☐ MRI-based neuroimaging

Antibodies

Antibodies used	All antibodies were gene synthesized at GeneArt from available sequences and produced recombinantly in our laboratory.
Validation	Binding of each of the antibodies to PDL1 receptor was tested by biolayer interferometry. Binding concentrations are indicated in Material and Methods.

Eukaryotic cell lines

Policy information about cell lines and Sex and Gender in Research

Cell line source(s)	FreeStyle™ 293-F, COLO205, NCI-H2122, NCI-H2228 and MCF7 cells purchased from ATCC
Authentication	Certificate provided by manufacturer.
Mycoplasma contamination	Certificate provided by manufacturer.
Commonly misidentified lines (See ICLAC register)	No commonly misidentified cell lines were used in the study.

Animals and other research organisms

Policy information about studies involving animals; ARRIVE guidelines recommended for reporting animal research, and Sex and Gender in Research

Laboratory animals	8-week-old C.B-17/IcrHan®Hsd-Prkdcscid female mice
Wild animals	The study did not involve wild animals
Reporting on sex	As no sex-based differences in antibody stability were observed in our preliminary analyses, the experiment was performed using female subjects. Given the small group size (3 animals per group), this approach also helped minimize animal use.
Field-collected samples	The study did not involve samples collected from the field
Ethics oversight	Animal protocols were approved by the Animal Research Ethics Board of CICbioGUNE (codes P-CBG-CBBA-0121 and P-CBG-CBBA-1321) and the Competent Authority (Diputación de Bizkaia) according to the guidelines of the European Union Council (Directive 2010/63/EU) and Spanish Government regulations (RD 53/2013).

Note that full information on the approval of the study protocol must also be provided in the manuscript.

Plants

Seed stocks	*Report on the source of all seed stocks or other plant material used. If applicable, state the seed stock centre and catalogue number. If plant specimens were collected from the field, describe the collection location, date and sampling procedures.*
Novel plant genotypes	*Describe the methods by which all novel plant genotypes were produced. This includes those generated by transgenic approaches, gene editing, chemical/radiation-based mutagenesis and hybridization. For transgenic lines, describe the transformation method, the number of independent lines analyzed and the generation upon which experiments were performed. For gene-edited lines, describe the editor used, the endogenous sequence targeted for editing, the targeting guide RNA sequence (if applicable) and how the editor was applied.*
Authentication	*Describe any authentication procedures for each seed stock used or novel genotype generated. Describe any experiments used to assess the effect of a mutation and, where applicable, how potential secondary effects (e.g. second site T-DNA insertions, mosiacism, off-target gene editing) were examined.*

Flow Cytometry

Plots

Confirm that:

☒ The axis labels state the marker and fluorochrome used (e.g. CD4-FITC).

☒ The axis scales are clearly visible. Include numbers along axes only for bottom left plot of group (a 'group' is an analysis of identical markers).

☒ All plots are contour plots with outliers or pseudocolor plots.

☒ A numerical value for number of cells or percentage (with statistics) is provided.

Methodology

Sample preparation	Colo205 (ATCC) cells were harvested in fresh RPMI supplemented with 10% FBS and counted. 50.000 cells/well were seeded in 200 µL in V- bottom 96-well plates. 2 µL of peptides ranging from 20 to 0 µM (2-fold dilutions) were added to the cells and incubated at 37° C, 5% CO_2, and 70% humidity for 30 minutes. Then, plates were centrifuged at 1250 rpm for 5 minutes, the supernatant was discarded, and cells were resuspended in 200 µL of PBS containing 7-AAD (7-aminoactinomicina D, Thermo Fisher Scientific) according to the manufacturer's instructions.
Instrument	CytoFlex S Cytometer (BC01011), Beckman Coulter.
Software	CytExpert Software
Cell population abundance	Not applicable. No sorting have been done in this study.
Gating strategy	Cells are gated based on FSC vs SSC to select populations with specific size and granularity and doublets are excluded using SSC-A vs SSC-H gating. Permeabilized cells stain positive with the 7-AAD probe (detected by APC laser on the cytometer) and exhibit a shift in APC intensity compared to non-permeabilized (healthy) cells. Percentage of permeabilized cells is subtracted from this plot for each condition.

☒ Tick this box to confirm that a figure exemplifying the gating strategy is provided in the Supplementary Information.
